# Supplementary material for: Twelve-month prevalence of haemarthrosis and joint disease using the Haemophilia Joint Health score: evaluation of the UK National Haemophilia Database and Haemtrack patient reported data: an observational study
Source: BMJ Open. 2022 Jan 12;12(1):e052358. doi: 10.1136/bmjopen-2021-052358 (PMC8756269; doi:10.1136/bmjopen-2021-052358)
Supplement: Author's manuscript [file bmjopen-2021-052358.draft_revisions.pdf]

BMJ Open

BMJ Open is committed to open peer review. As part of this commitment we make the peer review history of every article we publish publicly available.

When an article is published we post the peer reviewers' comments and the authors' responses online. We also post the versions of the paper that were used during peer review. These are the versions that the peer review comments apply to.

The versions of the paper that follow are the versions that were submitted during the peer review process. They are not the versions of record or the final published versions. They should not be cited or distributed as the published version of this manuscript.

BMJ Open is an open access journal and the full, final, typeset and author-corrected version of record of the manuscript is available on our site with no access controls, subscription charges or pay-per-view fees (http://bmjopen.bmj.com).

If you have any questions on BMJ Open's open peer review process please email info.bmjopen@bmj.com

BMJ Open

Twelve month prevalence of haemarthrosis and joint disease using the haemophilia joint health score; evaluation of the UK National Haemophilia Database and Haemtrack patient reported data

Journal:	*BMJ Open*
Manuscript ID	bmjopen-2021-052358
Article Type:	Original research
Date Submitted by the Author:	14-Apr-2021

Complete List of Authors:	Wilkins, Richard; University of Leeds Leeds Institute of Rheumatic and Musculoskeletal Medicine; Leeds Teaching Hospitals NHS Trust, Leeds haemophilia Comprehensive Care Centre<br>Stephensen, David; East Kent Hospitals University NHS Foundation Trust, Haemophilia Centre; Barts Health NHS Trust, Haemophilia Centre<br>Siddle, Heidi; University of Leeds, Leeds Institute of Rheumatic and Musculoskeletal Medicine<br>Scott, Martin; Manchester Royal Infirmary, University Department of Clinical Haematology,; The University of Manchester, Institute of Cancer Sciences, Faculty of Biology, Medicine and Health<br>Xiang, Hua; UKHCDO National Haemophilia Database<br>Horn, Elizabeth; Leeds Teaching Hospitals NHS Trust, Leeds haemophilia Comprehensive Care Centre<br>Palmer, Ben; UKHCDO National Haemophilia Database<br>Chapman, Graham; University of Central Lancashire, School of Sport and Health Sciences<br>Richards, Michael ; Leeds Teaching Hospitals NHS Trust, Leeds haemophilia Comprehensive Care Centre<br>Walwyn, Rebecca; University of Leeds, Clinical Trials Research Unit, Leeds Institute of Clinical Trials Research<br>Redmond, Anthony; University of Leeds, Leeds Institute of Rheumatology and Musculoskeletal Medicine; Leeds Teaching Hospitals NHS Trust, NIHR Leeds Biomedical Research Centre

Keywords:	Bleeding disorders & coagulopathies < HAEMATOLOGY, Foot & ankle < ORTHOPAEDIC & TRAUMA SURGERY, Musculoskeletal disorders < ORTHOPAEDIC & TRAUMA SURGERY

SCHOLARONE™
Manuscripts

I, the Submitting Author has the right to grant and does grant on behalf of all authors of the Work (as defined in the below author licence), an exclusive licence and/or a non-exclusive licence for contributions from authors who are: i) UK Crown employees; ii) where BMJ has agreed a CC-BY licence shall apply, and/or iii) in accordance with the terms applicable for US Federal Government officers or employees acting as part of their official duties; on a worldwide, perpetual, irrevocable, royalty-free basis to BMJ Publishing Group Ltd ("BMJ") its licensees and where the relevant Journal is co-owned by BMJ to the co-owners of the Journal, to publish the Work in this journal and any other BMJ products and to exploit all rights, as set out in our licence.

The Submitting Author accepts and understands that any supply made under these terms is made by BMJ to the Submitting Author unless you are acting as an employee on behalf of your employer or a postgraduate student of an affiliated institution which is paying any applicable article publishing charge ("APC") for Open Access articles. Where the Submitting Author wishes to make the Work available on an Open Access basis (and intends to pay the relevant APC), the terms of reuse of such Open Access shall be governed by a Creative Commons licence – details of these licences and which Creative Commons licence will apply to this Work are set out in our licence referred to above.

Other than as permitted in any relevant BMJ Author's Self Archiving Policies, I confirm this Work has not been accepted for publication elsewhere, is not being considered for publication elsewhere and does not duplicate material already published. I confirm all authors consent to publication of this Work and authorise the granting of this licence.

For peer review only

Twelve-month prevalence of haemarthrosis and joint disease using the haemophilia joint health score; evaluation of the UK National Haemophilia Database and Haemtrack patient reported data.

Richard A Wilkins[1,2], David Stephensen [3,4] Heidi J Siddle[1,], Martin J. Scott[5,6], Hua Xiang[7,] Elizabeth Horn[2], Ben Palmer[7], Graham J Chapman[8], Michael Richards[2], Rebecca Walwyn[9], Anthony C Redmond [1,10].

1. Leeds Institute of Rheumatic and Musculoskeletal Medicine (LIRMM), University of Leeds, Leeds, UK
2. Leeds haemophilia Comprehensive Care Centre, Leeds Teaching Hospitals NHS trust, Leeds, UK
3. The Royal London Hospital Haemophilia Centre, Barts and The London School of Medicine and Dentistry, Queen Mary University London, London, UK
4. Kent Haemophilia & Thrombosis Centre, East Kent Hospitals University NHS Trust, Canterbury, UK
5. University Department of Clinical Haematology, Manchester Royal Infirmary, Manchester, UK
6. Institute of Cancer Sciences, Faculty of Biology, Medicine and Health, The University of Manchester, Manchester, UK
7. UKHCDO National Haemophilia Database, Manchester, UK
8. School of Sport and Health Sciences, University of Central Lancashire, Preston, UK
9. Clinical Trials Research Unit, Leeds Institute of Clinical Trials Research, University of Leeds, Leeds, UK
10. NIHR Leeds Biomedical Research Centre, Leeds Teaching Hospitals NHS Trust, Leeds, UK

For peer review only

Correspondence:

Richard A Wilkins, Section of Clinical Biomechanics and Physical Medicine, Leeds Institute of Rheumatic and Musculoskeletal Medicine, University of Leeds, Leeds, UK

Email: r.a.wilkins@leeds.ac.uk

Key words: Prevalence, haemarthrosis, haemarthropathy, annualised joint bleed rate, haemophilia joint health score, prophylaxis

For peer review only

Abstract (300 words) for BMJ open

Objectives: To report the 12 month prevalence of joint bleeding and joint disease status at an individual joint level, in children and adults with severe haemophilia A and B without a current inhibitor**.**

Methods and analysis: Application was made to the UK National Haemophilia Database (NHD) with a request for the following; Haemtrack patient reported treatment record and Haemophilia Joint Health Scores (HJHS) in children (<18y) and adults (≥18y) with severe haemophilia A (HA) and B (HB) (FVIII/FIX, <0.01 iu/ml) without a current inhibitor. Data were collated and reported for 1st January to 31st December 2018.

Results: 2238 cases were identified, 463 patients had fully itemised HJHS of whom 273 were Haemtrack compliant. The median (IQR) age of children was 10 (6-13) and adults 40 (29-50) years. Haemarthrosis prevalence in HA/HB children was 33% and 47%, respectively and 60% and 42%, respectively, in adults. The most common site of haemarthrosis in children was the knee in HA and ankle in HB. In adults, the incidence of haemarthrosis at the ankles and elbows was equal. Median (IQR) total HJHS in HA/HB children were 0 (0;0). In adults with HA/HB, HJHS were 18 (6; 31) and 11 (5; 24), respectively. In adults with HA/HB, mean (SD) ankle HJHS of 3.8 (4.1) and median 4.0 (0.0; 8.0) were higher than the knee (mean 2.9 (4.1) and median 1.0 (0.0; 5.0) and elbow (mean 3.3 (4.1) and median 1.0 (0.0; 7.0) joints.

Conclusion; During 2018, NHD prevalence data for haemarthrosis indicate that only two-thirds of children and one-third of adults from a UK cohort compliant with prophylaxis were bleed free. Median HJHS of zero in children suggests joint disease status is either unaffected during childhood or undetected by the HJHS. In adults, moderately higher HJHS are reported for the ankles indicating worse joint health.

For peer review only - http://bmjopen.bmj.com/site/about/guidelines.xhtml

Strength and limitations of the study (methodology)

- This study reports the 12 month prevalence of haemarthrosis in children and adults with severe haemophilia without current inhibitors, and associated HJHS as a measure of joint disease
- Prevalence and site were collated retrospectively from Haemtrack and HJHS from the National Haemophilia Database
- Only the most compliant of patients who were adherent to taking and reporting prophylaxis on a national electronic treatment diary Haemtrack with concurrent HJHS scores were included
- Sample size was affected by methodology in including those with electronic fully itemised HJHS and above 75% threshold of compliance.
- The design of this study does not allow us to observe longitudinal joint bleed or joint health status.

For peer review only

Introduction:

Haemophilia is a rare x-linked recessive genetic disorder characterised by bleeding into soft tissue and joints [1]. The most common forms are haemophilia A and B, affecting 1:10000 and between 1:35,000 and 1:50,000 respectively. The disease is further characterised by the levels of factor VIII (FVIII) and factor VIX (FVIX), with the most severely affected having less than 1% (<0.01 IU/mL) circulating clotting factor (severe haemophilia) [2]. Musculoskeletal bleeding is the most common haemorrhagic manifestation, with 90% of bleeds occurring in muscles or joints [1]. The presence of blood products within the joint space and the process of removal leads to synovial hypertrophy, haemosiderin deposition and eventually arthropathic joint changes [3]. Over time, repeated haemarthrosis results in chronic synovitis, changes in cartilage and bone composition and progressive chronic haemarthropathy [4, 5].

Infusion of replacement clotting factor concentrates (CFC) is prescribed with the aim of elevating circulating factor to a level that halts spontaneous and traumatic bleeding [1]. CFC treatment is not without complication. The development of anti-Factor antibodies or "inhibitors" in some people produces an immune response to CFC infusion that significantly reduces the effectiveness of CFC treatment. Development of inhibitors increase the risk of bleeding, joint damage and requirement for factor treatment bypassing agents [6]. Ultimately the aim of modern treatment of haemophilia is prevention of joint bleeds with a target of achieving zero bleeds whenever possible. Prevention of haemarthrosis in all age groups is important and in particular in children, where musculoskeletal immaturity exposes joints to greater risk of damage in later life. Multiple studies have shown that early initiation of CFC prophylaxis in children delays joint damage and reduces joint disease [7-10]. In adults, multi-joint haemarthropathy remains a common feature of the disease, but even prophylaxis started in adulthood decreases bleeding, improves pain and improves health related QoL (HRQoL) [11]. Therefore in children and adults prophylaxis is considered the standard of care for all patients [11, 12]. Traditionally, prophylactic treatment in severe haemophilia aims to maintain factor VIII (FVIII) or Factor IX (FIX) at a trough level >0.01 iu/ml. It is apparent that many patients

For peer review only

experience spontaneous as well as traumatic bleeds, despite achieving trough factor levels > 0.01 iu/ml. Several approaches have been adopted or are being investigated with the aim of attaining complete bleed avoidance, including more individualised treatment with standard half-life products, the use of coagulation factors with extended half-lives, and innovative non-factor treatments [12-15].

Recent evaluation of real world treatment regimes in severe and moderate haemophilia in the UK and Europe, has shown that despite adequate coagulation factor concentrate availability, treatment is still suboptimal. In 2015, data from the United Kingdom National Haemophilia Database (NHD) reported median (IQR) annualised bleed rates (ABR)/ annualised joint bleed rates (AJBR) in children (0-11y) and adolescents (12-18y) of 1.0 (0.0-0.5)/ 0.0 (0.0-1.0) and 2.0 (0.0-7.0)/ 1.0 (0.0-3.0), respectively. ABR in adults with severe haemophilia A on prophylaxis were 2.0 (IQR 0.0-7.0) and AJBR was 1.0 (IQR 0.0-4.0) with only 29% bleed free and 34% joint bleed free [16]. Similarly, reported European (Belgium, France, Germany, Italy, Spain, Sweden, and UK) data shows median AJBR of 1.0 – 4.0. [16, 17]. However, data on bleeding frequency and severity of haemarthropathy at an individual joint level is lacking.

The main sites of haemarthrosis are the elbows, ankles and the knees, with the shoulders, wrists and hips less commonly affected and data for these sites not collated by the NHD or recorded as part of the HJHS [18]. Incidence of haemarthrosis and joint disease at an individual joint level are unknown. Those deemed most compliant with prophylaxis are less likely to experience repeated incidents of haemarthrosis and therefore less likely to have established joint disease when compared to those who do not adhere to treatment. This may be a smaller proportion than those who do not adhere to treatment but these cases are important in gauging the efficacy of current treatments [11, 19, 20]. Understanding prevalence and joint disease in the most compliant of patients may provide direction for future research of patient compliance and management of joint disease, including non-pharmacological interventions and intra-articular therapies commonly used in the management of MSK conditions.

For peer review only

Objective

The primary objective of this study is to determine the prevalence and incidence of joint bleeding and joint disease using the HJHS at an individual joint level in children and adults with severe haemophilia A and B without a current inhibitor.

For peer review only

Methods:

Ethical approval was obtained on 24th January 2017 (IRAS: 206141, R&D: PD16/227) Approval to access data from the UKHCDO NHD Data Analysis Group was granted on 12th July 2019 and the analysis report produced by UKHCDO on the 4th October 2019. The study has been reported in accordance with the UKHCDO NHD guidelines and regulations.

Data on bleed prevalence and site were collated retrospectively from Haemtrack and Haemophilia Joint Health Scores from the National Haemophilia Database. Haemtrack is a UK national online treatment diary in which individual patients regularly report details of treatments with coagulation factor concentrates (CFC) [20, 21]. Details of home delivery of CFC treatment to patients is recorded by the corresponding haemophilia treatment centre and then uploaded to the NHD. When CFC is administered by the patient that individual treatment is then recorded on Haemtrack, including the reason for each treatment such as prophylaxis or bleed treatment and the site of each bleed. Data recorded in Haemtrack are then integrated with NHD [20]. The 2018-2019 UKHCDO report indicated median compliance at haemophilia comprehensive care centres (CCC) and haemophilia treatment centres (HC) of 90% and 93% respectively [22]. The HJHS is a measure of joint health status in patients with haemophilia and forms part of the UKHCDO haemophilia management guidelines [23, 24]. The HJHS Version 2.1 is collated as six individual joint scores (0-20) and compiled with a global gait score (0-4) to a total score (0-124). A higher HJHS score represents worse joint health.

Participants were children (<18 years old) and adults (≥18 years old) with severe haemophilia A and B (FVIII or FIX <0.01 IU/mL) without a current inhibitor, who had been issued with coagulation factor concentrates in the UK between 1st January and 31st December 2018. Regular prophylaxis was defined for those using standard half-life (SHL) prophylaxis as >=2 infusions per week for Haemophilia A, and >=1 infusions/week for haemophilia B for >45 weeks/year; for patients using extended half-life (EHL) products, >=1 infusions/week for haemophilia A, and more than once every two weeks for haemophilia B for >45 weeks/year. Low dose prophylaxis is not prescribed in the UK, therefore, prophylaxis was assumed as

For peer review only

above 25 IU/kg to maintain a trough level above 0.02 IU/ml [25]. Those included in the analysis were Haemtrack compliant (defined as recorded use of ≥75% of received factor concentrate) with a corresponding electronically recorded Haemophilia Joint Health Score (HJHS) Version 2.1.

The joint bleed prevalence (%) for paediatric and adult patients and AJBR and HJHS were collated from Haemtrack and NHD. AJBR were reported by patients through the Haemtrack and recorded over the 12-month study period (1st January to 31st December 2018). Adequate primary and secondary prophylaxis and adherence to treatment are known to reduce bleed rates and reduce the burden of joint disease [11, 19]. Therefore only data from the most compliant patients (≥75% received factor concentrate vs recorded in Haemtrack) were reported as per the NHD standard operating procedure for data analysis and reporting. Joint bleed prevalence, AJBR and HJHS are reported for all joints (total) and in each individual joint. Data are summarised using means and standard deviations (SD) or medians and interquartile ranges (IQR, 25; 75 percentiles).

Patient and Public Involvement: Patients from the Leeds Haemophilia Comprehensive Care Centre, Leeds, UK and The NIHR Leeds Biomedical Research Centre (BRC), Leeds, UK were involved in the original design of the author's clinical doctoral research fellowship and this original article.

For peer review only

Results:

During 2018, 2238 individuals with severe haemophilia A (n=1889) and B (n=349) without a current inhibitor were registered with the NHD and 1396 were registered with Haemtrack. Electronically recorded fully itemised HJHS data was available for 463 patients with contemporaneous Haemtrack available for 273 individuals of whom 86.8% (n=237) had haemophilia A and 13.2% (n=36) haemophilia B. Participant age and treatment characteristics are presented in Table 1.

Table 1: Participant characteristics

Patient characteristics	Haemophilia A		Haemophilia B	
	Age < 18 (n=80)	Age ≥ 18 (n=157)	Age < 18 (n=17)	Age ≥ 18 (n=19)
Age (median, IQR)	10 (7; 13)	40 (29; 50)	12 (7;14)	45 (25; 48)
SHL	67% (n=54)	77% (n=121)	18% (n=3)	32%(n=6)
EHL	29% (n=23)	23% (n=36)	70% (n=12)	42%(n=8)
SHL-EHL	4% (n=3)	0%	12% (n=2)	26% (n=5)

SHL= Standard Half-life product, EHL= Extended Half-life product, SHL-EHL=switch from a SHL to a EHL during the 12 month study period.

Joint bleed prevalence and annualised bleed rate

Joint bleed prevalence and individual joint bleed incidence are categorised by age, haemophilia type and joint, and are presented in Table 2. Children with haemophilia A (32.5%) and haemophilia B (47.1%) reported at least one incidence of joint bleeding. Adults with haemophilia A (59.9%) and B (42.1%) reported at least one bleed over the same time period. Mean AJBR by ankles, knees, and elbows are presented in Figure 1.

Figure 1: Combined AJBR for children and adults with severe haemophilia A and B.

For peer review only

Table 2. Annual joint bleed prevalence and AJBR of children and adults

Annual Joint Bleeds			Haemophilia A		Haemophilia B	
Joint			Age < 18 (n=80)	Age ≥ 18 (n=157)	Age < 18 (n=17)	Age ≥ 18 (n=19)
All Joints	Joint bleed prevalence	n (%)	26 (32.5)	94 (59.9)	8 (47.1)	8 (42.1)
	Annual joint bleed Rate	Mean (SD)	0.81 (1.68)	3.90 (7.00)	1.00 (1.18)	2.04 (3.59)
		Median (IQR)	0.0 (0.0;1.0)	1.0 (0.0;4.4)	0.0 (0.0;2.0)	0.0 (0.0;3.5)
Right Ankle	Joint bleed prevalence	n (%)	2 (2.5)	27 (17.2)	1 (5.9)	2 (10.5)
	Annual joint bleed Rate	Mean (SD)	0.08 (0.57)	0.38 (1.06)	0.06 (0.24)	0.16 (0.51)
		Median (IQR)	0.0 (0.0;0.0)	0.0 (0.0;0.0)	0.0 (0.0;0.0)	0.0 (0.0;0.0)
Left ankle	Joint bleed prevalence	n (%)	5 (6.3)	35 (22.3)	5 (29.4)	2 (10.5)
	Annual joint bleed Rate	Mean (SD)	0.10 (0.44)	0.61 (1.98)	0.36 (0.62)	0.11 (0.33)
		Median (IQR)	0.0 (0.0;0.0)	0.0 (0.0;0.0)	0.0 (0.0;1.0)	0.0 (0.0;0.0)
Right knee	Joint bleed prevalence	n (%)	13 (16.3)	27 (17.2)	1 (5.9)	2 (10.5)
	Annual joint bleed Rate	Mean (SD)	0.20 (0.56)	0.41 (1.48)	0.18 (0.73)	0.53 (2.08)
		Median (IQR)	0.0 (0.0;0.0)	0.0 (0.0;0.0)	0.0 (0.0;0.0)	0.0 (0.0;0.0)
Left knee	Joint bleed prevalence	n (%)	7 (8.8)	24 (15.3)	1 (5.9)	2 (10.5)
	Annual joint bleed Rate	Mean (SD)	0.11 (0.39)	0.29 (0.96)	0.10 (0.42)	0.21 (0.72)
		Median (IQR)	0.0 (0.0;0.0)	0.0 (0.0;0.0)	0.0 (0.0;0.0)	0.0 (0.0;0.0)
Right elbow	Joint bleed prevalence	n (%)	6 (8.0)	29 (18.5)	1 (5.9)	3 (15.8)
	Annual joint bleed Rate	Mean (SD)	0.08 (0.27)	0.39 (1.12)	0.06 (0.24)	0.28 (0.78)
		Median (IQR)	0.0 (0.0;0.0)	0.0 (0.0;0.0)	0.0 (0.0;0.0)	0.0 (0.0;0.0)
Left elbow	Joint bleed prevalence	n (%)	4 (5.0)	35 (22.3)	1 (5.9)	2 (10.5)
	Annual joint bleed Rate	Mean (SD)	0.12 (0.73)	0.81 (2.38)	0.06 (0.24)	0.17 (0.53)
		Median (IQR)	0.0 (0.0;0.0)	0.0 (0.0;0.0)	0.0 (0.0;0.0)	0.0 (0.0;0.0)

Joint bleed prevalence (%): Numerator = number of patients who had bleeds, Denominator = total cohort number,

For peer review only

Haemophilia joint health score

HJHS categorised by age, haemophilia type and joint are presented in Table 3. Median (IQR) of HJHS in children were 0.0 (0.0; 0.0) in both haemophilia A and B. In adults the total HJHS were higher than in children; the total HJHS is higher in haemophilia A than haemophilia B. At an individual joint level both mean (SD) and median (IQR) ankle HJHS of 4.6 (4.3)/ 4.0 (0.0; 8.0) were higher than for the knee 2.9 (4.1)/ 1.00 (0.0; 5.0) and elbow 3.3 (4.1)/ 1.0 (0.0; 7.0).

Table 3. Haemophilia joint health scores for children and adults

Haemophilia joint health scores			Haemophilia A		Haemophilia B	
Joint			Age < 18 (n=80)	Age ≥ 18 (n=157)	Age < 18 (n=17)	Age ≥ 18 (n=19)
All Joints	HJHS (total)	Mean (SD)	0.7 (1.0)	21.2 (16.8)	0.4 (0.9)	15.4 (15.1)
		Median (IQR)	0.0 (0.0;0.0)	18.0 (6.0;31.0)	0.0 (0.0;0.0)	11.0 (5.0;24.0)
Right Ankle	HJHS (total)	Mean (SD)	0.1 (0.4)	4.6 (4.2)	0.0 (0.0)	3.6 (4.1)
		Median (IQR)	0.0 (0.0;0.0)	4.0 (0.0;8.0)	0.0 (0.0;0.0)	2.0 (0.0;7.0)
Left ankle	HJHS (total)	Mean (SD)	0.0 (0.1)	4.6 (4.3)	0.3 (0.8)	4.8 (4.1)
		Median (IQR)	0.0 (0.0;0.0)	4.0 (0.0;8.0)	0.0 (0.0;0.0)	4.0 (1.0;8.0)
Right knee	HJHS (total)	Mean (SD)	0.2 (0.5)	2.7 (3.9)	0.0 (0.0)	2.5 (4.6)
		Median (IQR)	0.0 (0.0;0.0)	1.0 (0.0;4.0)	0.0 (0.0;0.0)	0.0 (0.0;1.0)
Left knee	HJHS (total)	Mean (SD)	0.1 (0.3)	2.9 (4.1)	0.1 (0.2)	1.3 (2.2)
		Median (IQR)	0.0 (0.0;0.0)	1.00 (0.0;5.0)	0.0 (0.0;0.0)	0.0 (0.0;2.0)
Right elbow	HJHS (total)	Mean (SD)	0.1 (0.7)	3.3 (4.1)	0.0 (0.0)	1.3 (2.6)
		Median (IQR)	0.0 (0.0;0.0)	1.0 (0.0;7.0)	0.0 (0.0;0.0)	0.0 (0.0;1.0)
Left elbow	HJHS (total)	Mean (SD)	0.2 (1.2)	3.2 (4.2)	0.1 (0.2)	2.1 (4.0)
		Median (IQR)	0.0 (0.0;0.0)	1.0 (0.0;6.0)	0.0 (0.0;0.0)	0.0 (0.0;1.0)

HJHS: Global Gait score not included

For peer review only

Discussion:

In this study we report the current prevalence of haemarthrosis in children and adults with severe haemophilia without current inhibitors, and associated HJHS as a measure of joint disease. The study was conducted retrospectively, using data from 2018 in a national database. In a national cohort of 2338 individuals, 463 patients had electronically-recorded fully itemised HJHS, with the sample size further reduced to 273 patients who met the fully Haemtrack compliant criteria. During the data collection period, 62% of the national cohort used HT, 20% of whom fulfilled compliance criteria set by the NHD, permitting analysis of haemarthrosis and joint health status of a representative sample of UK with severe haemophilia without inhibitors. The sample size whilst small is focused only the most compliant of patients and provides insight to the current compliance rates and reporting of joint diseases to the NHD. The results presented in this paper represent the likely best case scenario for the most complaint cases and this further highlight the 80% of patients who fail to either record or comply with treatment and raises questions as to the real compliance and adherence to treatment, as well as the concurrent joint disease in patients who do not meet the 75% NHD inclusion threshold.

In children with severe haemophilia, average AJBR were low across haemophilia types. One in three children did however experience a joint bleed during the 12-month data collection period. The majority of those included would have typically been provided prophylaxis from an early age and continue to adhere to a prophylaxis regime, but 30% of children still experienced haemarthrosis during the 12 month data collection period. HJHS itemised by joint were very low in children (Table 3) suggesting either minimal joint disease or that the HJHS might not be sensitive to early joint changes following haemarthrosis. Reliability of the HJHS has been explored in children and young adults and is reported to be sensitive to early joint changes [23, 26] , although individual joint HJHS of less than three at the knee and ankle are less able to identify pathological joint change when compared to MRI and US imaging [27]. Canine, mouse and human in-vitro models have demonstrated chondrocyte apoptosis and reduced

For peer review only

proteoglycan synthesis affecting cartilage matrix turnover within 48-96 hours of an induced joint bleed, suggesting a single joint bleed may have detrimental effects on joint cartilage [28-30]. Formally reported bleed rates in the NHD are relatively low, however micro bleeding (subclinical bleeding not clinically detectable, or experienced by the patient) is an emerging theme in haemophilia. Episodes of subclinical bleeding may contribute to the deterioration of joint health despite no clinically detectable signs of a joint bleed [3].

In the adult population, AJBR were higher than those reported in children, with mean (SD) AJBR of 3.9 (7.0) and median (IQR) 1.0 (0.0-4.4) in haemophilia A and 2.0 (3.6) and 0.0 (0.0-3.5) in haemophilia B, respectively. The 12-month prevalence was also higher, with 60% and 41% of adults with haemophilia A and B, respectively, experiencing at least one bleed over the period. HJHS scores at the ankle joint were similar to the elbows, with knees slightly less affected. Interestingly the median scores at both the knee and elbow were lower than that of the ankle, suggesting that there is worse ankle joint health overall when compared to other joints. Ankle joint changes are driven by the mechanical demand on the ankle and forces exerted on the joint during activities of daily living, in combination with structural and functional changes often seen in adolescents and adults with severe haemophilia [31, 32]. Our data suggest that very early signs of joint disease might not be detected by the HJHS; rather it measures the cumulative effect of haemarthropathy, not detectable until later years.

AJBR in this study are slightly lower (Table 2) than those reported in the UK THUNDER study conducted three years earlier using the same NHD database [11]. Scott et al. reported a median AJBR of 0.0 in children (0-11 years), 1.0 in adolescents (12-18 years) and 3.0 in adults aged 19 and above. Our prevalence data (Table 1) for both children and adults indicate a slight decrease AJBR since the Scott et al. study [16]. In terms of the treatment profile of those included in our study, about one quarter were now using an EHL product and 96% of those sampled are receiving and are compliant with treatment. In addition Scott et al did not include those patients with haemophilia B who are reported to have better joint health and less frequent joint bleeds [33]. Regardless, those sampled in this study still had up to four joint

For peer review only

bleeds over a 12-month period, with 60% of all adults reporting a minimum of one joint bleed. Forty percent of individuals sampled reported no bleeds and were well controlled, but for the remaining 60% it is unclear why joint bleeding occurred. Understanding why the 60% in this cohort reported haemarthrosis may lead to better targeted and individualised treatment and identification of other contributing factors such as lifestyle and altered, combined and individual joint biomechanics of the upper and lower limbs.

A limitation of this study is the low proportion of patients registered on the UK database that had full Haemtrack and itemised HJHS data recorded at the time of data collection. The NHD does not report bleed level data on patients who do not use Haemtrack owing to the difficulty in collecting data from paper diaries and established links at each haemophilia centre through the NHD Haemophilia Centre Information System (HCIS), limiting analysis to Haemtrack compliant users [20]. Bias may have been introduced by the study design through the inability to include those not recording treatment in Haemtrack and those for whom HJHS examinations were not reported or itemised by joint to the NHD. Although this is the largest reported dataset of HJHS, the lack of linkage between elements of the data limits its wider utility. As electronic reporting of HJHS to the NHD becomes more routine and the dataset expands, we will be able link HJHS and joint health to rates of haemarthrosis. Haemtrack data compliance is defined as ≥75% of home delivery treatment received being recorded as used by the patient and so those who met the inclusion criteria are regarded as "good reporters" and deemed likely to be compliant with treatment [20]. The current bleeding and joint disease profiles of those who receive and record treatment, but fall below the 75% treatment adherence criteria is unknown. Access to individual treatment dose and trough levels were not available from the database and is acknowledged as a limitation of this study. Reporting of these data relies on haemophilia centres uploading real time data, including trough levels and up to date measurements of weight but requires access to patient's data and requires better reporting methods to be achievable. Understanding joint haemarthrosis in this subset of patients may provide further insight into the real-world prevalence of haemarthrosis. As expected due to the lower

For peer review only

prevalence, the sample of haemophilia B patients in this analysis is smaller than the haemophilia A cohort, and therefore differences in joint bleed prevalence and HJHS between patients with haemophilia A and B should be interpreted with caution. Those with haemophilia B may present with a milder bleeding phenotype than that of haemophilia A regardless of severity or treatment [33-35]. In addition people with haemophilia B may display less severe levels of haemarthropathy, with differences in the specific pathophysiological mechanisms of joint disease underlined by different rates of joint deterioration and severity [36]. Direct comparison between disease types is limited and therefore further research is needed to explore whether the lower bleed rates and better joint health in people with haemophilia B suggested in this study can be confirmed.

History of spontaneous and traumatic bleeding could not be separated, owing to data reporting methods within Haemtrack. Whilst prophylaxis protects against spontaneous bleeding there is still a proportion of these treatment compliant adults reporting up to four joint bleeds in the 12 month study period. Haemarthrosis may occur as individual joint events, but our data highlights the burden on overall joint disease. A previous history of developing inhibitors and a history of on-demand treatment now using secondary prophylaxis may predispose patients to higher levels of joint disease and greater risk of subsequent haemarthrosis [11]. Further research is required therefore to understand the bleeding profile and burden of disease in adults with established joint disease and previous inhibitor status.

A further limitation is between-centre variability in HJHS assessment [37]. HJHS data from different haemophilia centres may be subject to inter-centre scoring variability, although workshops have been conducted in the UK to decrease inter-centre variability in HJHS scoring. Furthermore, we are unable to confirm the influence of other factors such as the presence of co-morbid musculoskeletal conditions on HJHS data. UKHCDO NHD data was also requested from those with moderate disease but there was insufficient data to include in the analysis. Future comparison by disease severity (severe and moderate) may provide further insight of those most at risk of haemarthropathy.

For peer review only

Clinical implication and conclusion:

In a UK cohort of Haemtrack compliant patients with severe haemophilia and without a current inhibitor, only 70% of children and 30% of adults remained haemarthrosis free during 2018. Haemarthrosis was most likely to be reported in the knee joint in children with haemophilia A, the ankle joint in children with haemophilia B, the elbow and ankle joint in adults with haemophilia A, and the elbow joint in adults with haemophilia B. Overall higher HJHS were reported for the ankle joint compared to the knee and elbow, suggesting that the ankle joint is the most severely compromised joint in people with haemophilia.

Investigation of impact on function and potential interventions that lessen the burden of disease are warranted. Future clinical studies would also benefit from understanding the bleeding profiles of those who do not meet compliance criteria for Haemtrack or other database-linked bleed data to obtain the true prevalence of haemarthrosis and joint disease.

For peer review only

Abbreviations

ABR: Annual bleed rate

AJBR: Annual joint bleed rate

CFC: Clotting factor concentrate

EHL: Extended half life

HJHS: Haemophilia joint health score

NHD: National Haemophilia Database

SHL: Standard half-life

UKHCDO: United Kingdom Haemophilia Doctors Organisation

For peer review only

Declarations

Ethics approval and consent to participate

Ethical approval was obtained to allow access the National Haemophilia Database, anonymised data. This study was approved by London Queen Square Research Ethics Committee (16/LO/2251) and NHS Health research Authority (IRAS ID 206141). Individual participant consent was not applicable.

For peer review only

Consent for publication

Consent to publish this paper was obtained from the UKHCDO NHD.

Availability of data and materials

The data that support the findings of this study are available from The National Haemophilia Database (NHD) but restrictions apply to the availability of these data, which were used under license for the current study, and so are not publicly available. Data are however available from the corresponding author upon reasonable request and with permission of the NHD.

Competing interests

RAW has received registration fees and support for travel from Roche.

DS has received research funding from Sobi, CSL and Roche; consultancy and speakers fees from Sobi and Takeda

EH has received speaker fees from Roche, sponsorship for travel from Sobi.

MJS has received research funding from Bayer; consultancy and speakers fees from Sobi and Roche; registration fees and support for travel from Sobi, Pfizer and CSL.

HJS is a HEE/NIHR Senior Clinical lecturer and has received funding from NIHR who also funded this research.

ACR is a NIHR Senior Investigator and has received funding from NIHR who also funded this research.

GJC, RW, HX, BP and MR report no competing interests

Funding

RAW is funded by a National Institute for Health Research (NIHR) Clinical Doctoral Research Fellowship (ICA-CDRF-2015-01-0120); ACR is in part supported through the NIHR Leeds Biomedical Research Centre, Leeds, UK. This paper presents independent research funded by the National Institute for Health Research (NIHR). The views expressed are those of the authors and not necessarily those of the NHS, the NIHR or the Department of Health.

Author's contributions

The study was conceived by RAW, AR, GC, RW and HJS. Analysis was undertaken by members of staff at the NHD (HX and BP).The manuscript was written by RAW and DS. Subsequent drafts were edited and approved by RAW, DS, MJS, AR, GC, MR, EH, HJS, and RW.

Acknowledgements

The authors would like to thank the centre directors, physiotherapists and staff of all UK haemophilia centres for their hard work in collecting data and responding to data queries. We would also like to thank the members of the UKHCDO Data Analysis Group for advising on data interpretation; Prof Charles Hay, Prof Peter Collins, Dr Elizabeth Chalmers, Dr Ryan Cheal, Prof Pratima Chowdary, Simon Fletcher, Dr Georgina Hall, Dr Dan Hart, Dr Ri Liesner, Andrew McNally, Ben Palmer, Paul Sartain, Dr Martin Scott, Dr Susie Shapiro, David Stephensen, Dr Hua Xiang. The staff at the National Haemophilia Database are also thanked for their contribution to data collection.

Author's information

ORCID (those with ORCID IDs)

Richard Wilkins: 0000-0003-1885-5472

Anthony Redmond: 0000-0002-8709-9992

Heidi Siddle: 0000-0002-6015-332X

For peer review only

Graham Chapman: 0000-0003-3983-6641

Martin Scott: 0000-0002-0854-1309

David Stephensen: 0000-0002-6175-3343

Rebecca Walwyn: 0000-0001-9120-1438

For peer review only

References

1. Bolton-Maggs, P.H. and K.J. Pasi, *Haemophilias a and b.* The Lancet, 2003. **361**(9371): p. 1801-1809.
2. Biggs, R. and R.J.B.j.o.h. Macfarlane, *Haemophilia and related conditions: a survey of 187 cases.* 1958. **4**(1): p. 1-27.
3. Gringeri, A., B. Ewenstein, and A.J.H. Reininger, *The burden of bleeding in haemophilia: is one bleed too many?* Haemophilia, 2014. **20**(4): p. 459-463.
4. Van Vulpen, L., K. Holstein, and C.J.H. Martinoli, *Joint disease in haemophilia: Pathophysiology, pain and imaging.* Haemophilia, 2018. **24**: p. 44-49.
5. Roosendaal, G. and F. Lafeber, *Pathogenesis of haemophilic arthropathy.* Haemophilia, 2006. **12**(s3): p. 117-121.
6. Hanley, J., et al., *Guidelines for the management of acute joint bleeds and chronic synovitis in haemophilia.* Haemophilia, 2017.
7. Manco-Johnson, M.J., et al., *Prophylaxis versus episodic treatment to prevent joint disease in boys with severe hemophilia.* New England Journal of Medicine, 2007. **357**(6): p. 535-544.
8. Oldenburg, J., *Optimal treatment strategies for hemophilia: achievements and limitations of current prophylactic regimens.* Blood, 2015. **125**(13): p. 2038-44.
9. Gringeri, A., et al., *A randomized clinical trial of prophylaxis in children with hemophilia A (the ESPRIT Study).* Journal of Thrombosis and Haemostasis, 2011. **9**(4): p. 700-710.
10. Srivastava, A., et al., *Guidelines for the management of hemophilia.* 2013. **19**(1): p. e1-e47.
11. Collins, P., et al., *Efficacy and safety of secondary prophylactic vs. on-demand sucrose-formulated recombinant factor VIII treatment in adults with severe hemophilia A: results from a 13-month crossover study.* Journal of Thrombosis and Haemostasis, 2010. **8**(1): p. 83-89.
12. Skinner, M.W., et al., *Achieving the unimaginable: Health equity in haemophilia.* Haemophilia, 2019.
13. Skinner, M.W., *WFH: closing the global gap–achieving optimal care.* Haemophilia, 2012. **18**: p. 1-12.
14. Ebbert, P.T., et al., *Emicizumab prophylaxis in patients with haemophilia A with and without inhibitors.* Haemophilia, 2020. **26**(1): p. 41-46.
15. Chowdary, P.J.T.R., *Extended half-life recombinant products in haemophilia clinical practice–Expectations, opportunities and challenges.* Thrombosis Research, 2019.
16. Scott, M.J., et al., *Treatment regimens and outcomes in severe and moderate haemophilia A in the UK: The THUNDER study.* Haemophilia, 2019. **25**(2): p. 205-212.
17. Berntorp, E., et al., *European retrospective study of real-life haemophilia treatment.* Haemophilia, 2017. **23**(1): p. 105-114.
18. Stephensen, D., et al., *Changing patterns of bleeding in patients with severe haemophilia A.* Haemophilia, 2009. **15**(6): p. 1210-1214.
19. Thornburg, C.D., N.A.J.P.p. Duncan, and adherence, *Treatment adherence in hemophilia.* Patient preference adherence, 2017. **11**: p. 1677.
20. Hay, C., et al., *The haemtrack home therapy reporting system: Design, implementation, strengths and weaknesses: A report from UK Haemophilia Centre Doctors Organisation.* Haemophilia, 2017. **23**(5): p. 728-735.

For peer review only

21. UKHCDO. *united kingdom haemophilia centre doctors organisation*. 2020 [cited 2020 05/04/2020]; Available from: http://www.ukhcdo.org/nhd/.
22. UKHCDO, *UKHCDO Annual Report 2019, including bleeding disorder statistics* 2019.
23. Hilliard, P., et al., *Hemophilia joint health score reliability study.* Haemophilia, 2006. **12**(5): p. 518-525.
24. Ribeiro, T., et al., *Developing a new scoring scheme for the Hemophilia Joint Health Score 2.1.* Research and practice in thrombosis and haemostasis, 2019. **3**(3): p. 405-411.
25. Richards, M., et al., *A United Kingdom Haemophilia Centre Doctors' Organization guideline approved by the British Committee for Standards in Haematology: guideline on the use of prophylactic factor VIII concentrate in children and adults with severe haemophilia A.* 2010. **149**(4): p. 498-507.
26. Boehlen, F., L. Graf, and E.J.E.J.o.H. Berntorp, *Outcome measures in haemophilia: a systematic review.* 2014. **93**: p. 2-15.
27. Poonnoose, P., et al., *Correlating clinical and radiological assessment of joints in haemophilia: results of a cross sectional study.* Haemophilia, 2016. **22**(6): p. 925-933.
28. Hakobyan, N., et al., *Pathobiology of hemophilic synovitis I: overexpression of mdm2 oncogene.* Blood, 2004. **104**(7): p. 2060-2064.
29. Roosendaal, G., et al., *Blood-induced joint damage: a canine in vivo study.* Haemophilia, 1999. **42**(5): p. 1033-1039.
30. Hooiveld, M., et al., *Blood-induced joint damage: longterm effects in vitro and in vivo.* The Journal of rhematology, 2003. **30**(2): p. 339-344.
31. Pasta, G., et al., *Orthopaedic management of haemophilia arthropathy of the ankle.* Haemophilia, 2008. **14**(s3): p. 170-176.
32. Rodriguez-Merchan, E.C., *Management of hemophilic arthropathy of the ankle.* Cardiovascular and Hematological Disorders - Drug Targets, 2017. **17**(2): p. 111-118.
33. Mannucci, P. and M.J.H. Franchini, *Is haemophilia B less severe than haemophilia A?* Haemophilia, 2013. **19**(4): p. 499-502.
34. Santagostino, E., et al., *Severe hemophilia with mild bleeding phenotype: molecular characterization and global coagulation profile.* Journal of Thrombosis and Haemostasis

2010. **8**(4): p. 737-743.
35. Castaman, G. and D.J.h. Matino, *Hemophilia A and B: molecular and clinical characteristics of similar, but different diseases.* Haematologica

2019: p. haematol. 2019.221093.
36. Melchiorre, D., et al., *Clinical, instrumental, serological and histological findings suggest that hemophilia B may be less severe than hemophilia A.* Haematologica, 2016. **101**(2): p. 219-225.
37. Nijdam, A., et al., *Using routine Haemophilia Joint Health Score for international comparisons of haemophilia outcome: standardization is needed.* Haemophilia 2016. **22**(1): p. 142-147.

For peer review only

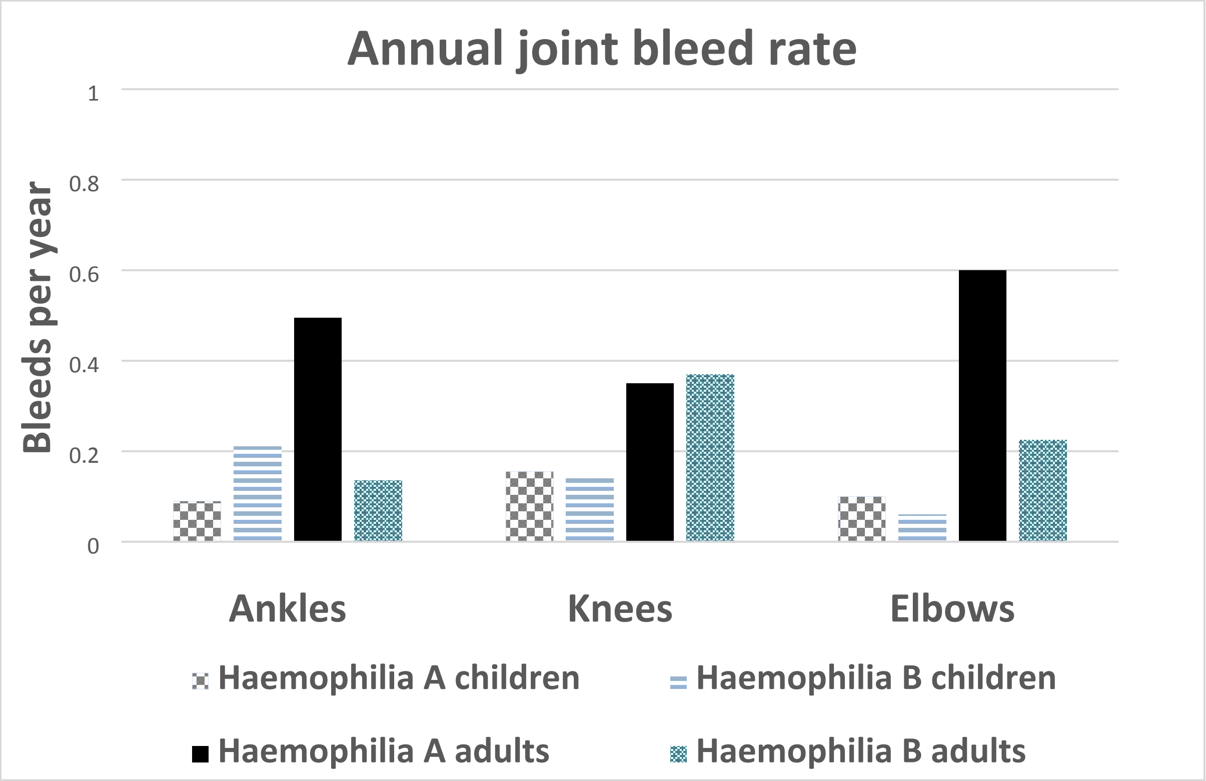

Figure 1: Combined AJBR for children and adults with severe haemophilia A and B.

For peer review only

BMJ Open

Twelve-month prevalence of haemarthrosis and joint disease using the haemophilia joint health score; evaluation of the UK National Haemophilia Database and Haemtrack patient reported data: an observational study

Journal:	*BMJ Open*
Manuscript ID	bmjopen-2021-052358.R1
Article Type:	Original research
Date Submitted by the Author:	02-Sep-2021
Complete List of Authors:	Wilkins, Richard; University of Leeds Leeds Institute of Rheumatic and Musculoskeletal Medicine; Leeds Teaching Hospitals NHS Trust, Leeds haemophilia Comprehensive Care Centre<br>Stephensen, David; East Kent Hospitals University NHS Foundation Trust, Haemophilia Centre; Barts Health NHS Trust, Haemophilia Centre<br>Siddle, Heidi; University of Leeds, Leeds Institute of Rheumatic and Musculoskeletal Medicine<br>Scott, Martin; Manchester Royal Infirmary, University Department of Clinical Haematology,; The University of Manchester, Institute of Cancer Sciences, Faculty of Biology, Medicine and Health<br>Xiang, Hua; UKHCDO National Haemophilia Database<br>Horn, Elizabeth; Leeds Teaching Hospitals NHS Trust, Leeds haemophilia Comprehensive Care Centre<br>Palmer, Ben; UKHCDO National Haemophilia Database<br>Chapman, Graham; University of Central Lancashire, School of Sport and Health Sciences<br>Richards, Michael ; Leeds Teaching Hospitals NHS Trust, Leeds haemophilia Comprehensive Care Centre<br>Walwyn, Rebecca; University of Leeds, Clinical Trials Research Unit, Leeds Institute of Clinical Trials Research<br>Redmond, Anthony; University of Leeds, Leeds Institute of Rheumatology and Musculoskeletal Medicine; Leeds Teaching Hospitals NHS Trust, NIHR Leeds Biomedical Research Centre
**Primary Subject Heading**:	Haematology (incl blood transfusion)
Secondary Subject Heading:	Rehabilitation medicine
Keywords:	Bleeding disorders & coagulopathies < HAEMATOLOGY, Foot & ankle < ORTHOPAEDIC & TRAUMA SURGERY, Musculoskeletal disorders < ORTHOPAEDIC & TRAUMA SURGERY

I, the Submitting Author has the right to grant and does grant on behalf of all authors of the Work (as defined in the below author licence), an exclusive licence and/or a non-exclusive licence for contributions from authors who are: i) UK Crown employees; ii) where BMJ has agreed a CC-BY licence shall apply, and/or iii) in accordance with the terms applicable for US Federal Government officers or employees acting as part of their official duties; on a worldwide, perpetual, irrevocable, royalty-free basis to BMJ Publishing Group Ltd ("BMJ") its licensees and where the relevant Journal is co-owned by BMJ to the co-owners of the Journal, to publish the Work in this journal and any other BMJ products and to exploit all rights, as set out in our licence.

The Submitting Author accepts and understands that any supply made under these terms is made by BMJ to the Submitting Author unless you are acting as an employee on behalf of your employer or a postgraduate student of an affiliated institution which is paying any applicable article publishing charge ("APC") for Open Access articles. Where the Submitting Author wishes to make the Work available on an Open Access basis (and intends to pay the relevant APC), the terms of reuse of such Open Access shall be governed by a Creative Commons licence – details of these licences and which Creative Commons licence will apply to this Work are set out in our licence referred to above.

Other than as permitted in any relevant BMJ Author's Self Archiving Policies, I confirm this Work has not been accepted for publication elsewhere, is not being considered for publication elsewhere and does not duplicate material already published. I confirm all authors consent to publication of this Work and authorise the granting of this licence.

For peer review only

Twelve-month prevalence of haemarthrosis and joint disease using the haemophilia joint health score; evaluation of the UK National Haemophilia Database and Haemtrack patient reported data: an observational study

Richard A Wilkins[1,2], David Stephensen [3,4] Heidi J Siddle[1,], Martin J. Scott[5,6], Hua Xiang[7,] Elizabeth Horn[2], Ben Palmer[7], Graham J Chapman[8], Michael Richards[2], Rebecca Walwyn[9], Anthony C Redmond [1,10].

1. Leeds Institute of Rheumatic and Musculoskeletal Medicine (LIRMM), University of Leeds, Leeds, UK
2. Leeds haemophilia Comprehensive Care Centre, Leeds Teaching Hospitals NHS trust, Leeds, UK
3. The Royal London Hospital Haemophilia Centre, Barts and The London School of Medicine and Dentistry, Queen Mary University London, London, UK
4. Kent Haemophilia & Thrombosis Centre, East Kent Hospitals University NHS Trust, Canterbury, UK
5. University Department of Clinical Haematology, Manchester Royal Infirmary, Manchester, UK
6. Institute of Cancer Sciences, Faculty of Biology, Medicine and Health, The University of Manchester, Manchester, UK
7. UKHCDO National Haemophilia Database, Manchester, UK
8. School of Sport and Health Sciences, University of Central Lancashire, Preston, UK
9. Clinical Trials Research Unit, Leeds Institute of Clinical Trials Research, University of Leeds, Leeds, UK
10. NIHR Leeds Biomedical Research Centre, Leeds Teaching Hospitals NHS Trust, Leeds, UK

For peer review only

For peer review only - http://bmjopen.bmj.com/site/about/guidelines.xhtml

Correspondence:

Richard A Wilkins, Section of Clinical Biomechanics and Physical Medicine, Leeds Institute of Rheumatic and Musculoskeletal Medicine, University of Leeds, Leeds, UK

Email: r.a.wilkins@leeds.ac.uk

Key words: Prevalence, haemarthrosis, haemarthropathy, annualised joint bleed rate, haemophilia joint health score, prophylaxis

For peer review only

Abstract (300 words) for BMJ open

Objectives: To report the 12 month prevalence of joint bleeds from the national haemophilia database (NHD) and Haemtrack, a patient-reported online treatment diary; and concurrent joint disease status using the haemophilia joint health score (HJHS) at individual joint level, in children and adults with severe haemophilia A and B without a current inhibitor**.**

Design: A 2018 retrospective database study of NHD from which 2238 cases were identified, 463 patients had fully itemised haemophilia joint health scores (HJHS) of whom 273 were compliant in recording treatment using Haemtrack.

Setting: England, Wales and Scotland, UK.

Participants: Children (<18y) and adults (≥18y) with severe haemophilia A (HA) and B (HB) (FVIII/FIX, <0.01 iu/ml) without a current inhibitor.

Primary and secondary outcomes: Prevalence of joint haemarthrosis, and concurrent joint health measured using the Haemophilia Joint Health Scores (HJHS).

Results: The median (IQR) age of children was 10 (6-13) and adults 40 (29-50) years. Haemarthrosis prevalence in HA/HB children was 33% and 47%, respectively and 60% and 42%, respectively, in adults. The most common site of haemarthrosis in children was the knee in HA and ankle in HB. In adults, the incidence of haemarthrosis at the ankles and elbows was equal. The median total HJHS in HA/HB children was 0 and in adults with HA/HB, were 18 and 11 respectively. In adults with HA/HB, the median ankle HJHS of 4.0 was higher than the median HJHS of 1.0 for both the knee and elbow.

Conclusion; Despite therapeutic advances, only two-thirds of children and one-third of adults were bleed-free, even in a UK cohort selected for high compliance with prophylaxis. The median HJHS of zero in children suggests joint health is relatively unaffected during childhood. In adults, bleed rates were highest in ankles and elbows, but the ankles led to substantially worse joint health scores.

For peer review only

Strength and limitations of the study (methodology)

- This study reports the 12 month prevalence of haemarthrosis in children and adults with severe haemophilia without current inhibitors, and associated HJHS as a measure of joint disease
- Prevalence and site were collated retrospectively from Haemtrack and HJHS from the National Haemophilia Database
- Only the most compliant of patients who were adherent to taking and reporting prophylaxis on a national electronic treatment diary Haemtrack with concurrent HJHS scores were included
- Sample size was affected by methodology including those with electronic fully itemised HJHS and above 75% threshold of compliance**.**
- The design of this study does not allow examination of longitudinal joint bleed or joint health status.

For peer review only

Introduction:

Haemophilia is a rare x-linked recessive genetic disorder characterised by bleeding into soft tissue and joints [1]. The most common forms are haemophilia A and B, affecting 1:10000 and between 1:35,000 and 1:50,000 respectively. The disease is further characterised by the levels of factor VIII (FVIII) and factor VIX (FVIX), with the most severely affected having less than 1% (<0.01 IU/mL) circulating clotting factor (severe haemophilia) [2]. Musculoskeletal bleeding is the most common haemorrhagic manifestation, with 90% of bleeds occurring in muscles or joints [1]. The presence of blood products within the joint space and the process of removal leads to synovial hypertrophy, haemosiderin deposition and eventually arthropathic joint changes [3]. Over time, repeated haemarthrosis results in chronic synovitis, changes in cartilage and bone composition and progressive chronic haemarthropathy [4, 5].

Infusion of replacement clotting factor concentrates (CFC) is prescribed with the aim of elevating circulating factor to a level that halts spontaneous and traumatic bleeding [1]. CFC treatment is not without complication. The development of anti-Factor antibodies or “inhibitors” in some people produces an immune response to CFC infusion that significantly reduces the effectiveness of CFC treatment. Development of inhibitors increase the risk of bleeding, joint damage and requirement for factor treatment bypassing agents [6]. Ultimately the aim of modern treatment of haemophilia is prevention of joint bleeds with a target of achieving zero bleeds whenever possible. Prevention of haemarthrosis in all age groups is important and in particular in children, where musculoskeletal immaturity exposes joints to greater risk of damage in later life. Multiple studies have shown that early initiation of CFC prophylaxis in children delays joint damage and reduces joint disease [7-10]. In adults, multi-joint haemarthropathy remains a common feature of the disease, but even prophylaxis started in adulthood decreases bleeding, improves pain and improves health related quality of life (HRQoL) [11]. Therefore in children and adults prophylaxis is considered the standard of care for all patients [11, 12]. Traditionally, prophylactic treatment in severe haemophilia aims to maintain Factor VIII (FVIII) or Factor IX (FIX) at a trough level >0.01 iu/ml. It is apparent that

For peer review only - http://bmjopen.bmj.com/site/about/guidelines.xhtml

many patients experience spontaneous as well as traumatic bleeds, despite achieving trough Factor levels > 0.01 iu/ml. Several approaches have been adopted or are being investigated with the aim of attaining complete bleed avoidance, including more individualised treatment with standard half-life products, the use of coagulation factors with extended half-lives, and innovative non-Factor treatments [12-15].

Recent evaluation of real world treatment regimes in severe and moderate haemophilia in the UK and Europe, has shown that despite adequate coagulation factor concentrate availability, treatment is still suboptimal. In 2015, data from the United Kingdom National Haemophilia Database (NHD) reported median (IQR) annualised bleed rates (ABR)/ annualised joint bleed rates (AJBR) in children (0-11y) and adolescents (12-18y) of 1.0 (0.0-0.5)/ 0.0 (0.0-1.0) and 2.0 (0.0-7.0)/ 1.0 (0.0-3.0), respectively. ABR in adults with severe haemophilia A on prophylaxis were 2.0 (IQR 0.0-7.0) and AJBR was 1.0 (IQR 0.0-4.0) with only 29% bleed free and 34% joint bleed free [16]. Similarly, reported European (Belgium, France, Germany, Italy, Spain, Sweden, and UK) data shows median AJBR of 1.0 – 4.0. [16, 17]. However, data on bleeding frequency and severity of haemarthropathy at an individual joint level is lacking.

The main sites of haemarthrosis are the elbows, ankles and the knees, with the shoulders, wrists and hips less commonly affected and data for these sites not collated by the NHD. The haemophilia joint health score (HJHS) is a standardised clinical assessment tool developed to assess upper and lower limb joint health status. The clinical assessments undertaken by specialist physiotherapists at 6-12 month intervals include measurement of swelling, alignment, range of motion, and muscle atrophy, and forms part of the UKHCDO haemophilia management guidelines [18, 19]. The HJHS is the most widely used score of joint health in haemophilia and has shown good to moderate correlations with radiological scores of joint disease using the Pettersson score [18]. However haemarthrosis is not reported by the HJHS and therefore incidence of haemarthrosis and joint disease at an individual joint level are unknown [20].

For peer review only - http://bmjopen.bmj.com/site/about/guidelines.xhtml

Those deemed most compliant with prophylaxis are less likely to experience repeated incidents of haemarthrosis and therefore less likely to have established joint disease when compared to those who do not adhere to treatment. This may be a smaller proportion than those who do not adhere to treatment but these cases are important in gauging the efficacy of current treatments [11, 19, 20]. Understanding prevalence and joint disease in the most compliant of patients may provide direction for future research of patient compliance and management of joint disease, including non-pharmacological interventions and intra-articular therapies commonly used in the management of MSK conditions.

Objective

The primary objective of this study is to determine the prevalence and incidence of joint bleeding and joint disease using the HJHS at an individual joint level in children and adults with severe haemophilia A and B without a current inhibitor.

For peer review only - http://bmjopen.bmj.com/site/about/guidelines.xhtml

Methods:

Ethical approval was obtained on 24th January 2017 (IRAS: 206141, R&D: PD16/227) Approval to access data from the UKHCDO NHD Data Analysis Group was granted on 12th July 2019 and the analysis report produced by UKHCDO on the 4th October 2019. The study has been reported in accordance with the UKHCDO NHD guidelines and regulations.

Data on bleed prevalence and site were collated retrospectively from the Haemtrack patient therapy recording system and the clinical Haemophilia Joint Health Score from the National Haemophilia Database. Haemtrack is a UK national online treatment diary in which individual patients regularly report details of treatments with coagulation factor concentrates (CFC) [20, 21]. Details of home delivery of CFC treatment to patients is recorded by the corresponding haemophilia treatment centre and then uploaded to the NHD. When CFC is administered by the patient that individual treatment is then recorded on Haemtrack, including the reason for each treatment such as prophylaxis or bleed treatment and the site of each bleed. Data recorded in Haemtrack are then integrated with NHD [20]. The 2018-2019 UKHCDO report indicated median compliance at haemophilia comprehensive care centres (CCC) and haemophilia treatment centres (HC) of 90% and 93% respectively with the NHD definition of compliance recorded use of ≥75% of received factor concentrate [22]. The HJHS Version 2.1 is collated as six individual joint scores (0-20) and compiled with a global gait score (0-4) to a total score (0-124). A higher HJHS score represents worse joint health.

Participants were children (<18 years old) and adults (≥18 years old) with severe haemophilia A and B (FVIII or FIX <0.01 IU/mL) without a current inhibitor, who had been issued with coagulation factor concentrates in the UK between 1st January and 31st December 2018. Regular prophylaxis was defined for those using standard half-life (SHL) prophylaxis as >=2 infusions per week for Haemophilia A, and >=1 infusions/week for haemophilia B for >45 weeks/year; for patients using extended half-life (EHL) products, >=1 infusions/week for haemophilia A, and more than once every two weeks for haemophilia B for >45 weeks/year. Low dose prophylaxis is not prescribed in the UK, therefore, prophylaxis was assumed as

For peer review only - http://bmjopen.bmj.com/site/about/guidelines.xhtml

above 25 IU/kg to maintain a trough level above 0.02 IU/ml [23]. Those included in the analysis were Haemtrack compliant (defined as recorded use of ≥75% of received factor concentrate) with a corresponding electronically recorded Haemophilia Joint Health Score (HJHS) Version 2.1.

The joint bleed prevalence (%) for paediatric and adult patients and AJBR and HJHS were collated from Haemtrack and NHD. AJBR were reported by patients through the Haemtrack and recorded over the 12 month study period (1st January to 31st December 2018). Adequate primary and secondary prophylaxis and adherence to treatment are known to reduce bleed rates and reduce the burden of joint disease [11, 19]. Therefore only data from the most compliant patients (≥75% received factor concentrate vs recorded in Haemtrack) were reported as per the NHD standard operating procedure for data analysis and reporting. Joint bleed prevalence, AJBR and HJHS are reported for all joints (total) and in each individual joint. Data are summarised using means and standard deviations (SD) or medians and interquartile ranges (IQR, 25; 75 percentiles).

Patient and Public Involvement: Patients from the Leeds Haemophilia Comprehensive Care Centre, Leeds, UK and The NIHR Leeds Biomedical Research Centre (BRC), Leeds, UK were involved in the original design of the author's clinical doctoral research fellowship and this original article.

For peer review only - http://bmjopen.bmj.com/site/about/guidelines.xhtml

Results:

During 2018, 2238 individuals with severe haemophilia A (n=1889) and B (n=349) without a current inhibitor were registered with the NHD and 1396 were registered with Haemtrack. Electronically recorded fully itemised HJHS data was available for 463 patients with contemporaneous Haemtrack available for 273 individuals of whom 86.8% (n=237) had haemophilia A and 13.2% (n=36) haemophilia B. Participant age and treatment characteristics are presented in Table 1.

Table 1: Participant characteristics

Patient characteristics	Haemophilia A		Haemophilia B	
	Age < 18 (n=80)	Age ≥ 18 (n=157)	Age < 18 (n=17)	Age ≥ 18 (n=19)
Age (median, IQR)	10 (7; 13)	40 (29; 50)	12 (7;14)	45 (25; 48)
SHL	67% (n=54)	77% (n=121)	18% (n=3)	32% (n=6)
EHL	29% (n=23)	23% (n=36)	70% (n=12)	42% (n=8)
SHL-EHL	4% (n=3)	0%	12% (n=2)	26% (n=5)

SHL= Standard Half-life product, EHL= Extended Half-life product, SHL-EHL=switch from a SHL to a EHL during the 12 month study period.

Joint bleed prevalence and annual bleed rate

Joint bleed prevalence (%) and individual joint prevalence, and total AJBR are presented in Table 2. Bleed data are categorised by age, haemophilia type (A and B) and the most commonly effect joints (left and right) of the elbows, knees and ankles. Joint bleed prevalence in children with haemophilia A (32.5%) and haemophilia B (47.1%) reported at least one incidence of joint bleeding over the 12 month study period. Adults with haemophilia A (59.9%) and B (42.1%) reported at least one bleed over the same time period. Median AJBR at individual joints for children and adults were 0.0 (0.0;0.0) with the exception of the left ankle in children with haemophilia B (0.0;1.0). Mean AJBR for adults and children at the ankles, knees, and elbows are presented in Figure 1.

For peer review only - http://bmjopen.bmj.com/site/about/guidelines.xhtml

Figure 1: Combined annual joint bleed rate for children (vertical and horizontal black columns) and adults (solid grey and black columns) with severe haemophilia A and B.

For peer review only

For peer review only - http://bmjopen.bmj.com/site/about/guidelines.xhtml

1 **Table 2. Annual joint bleed prevalence and AJBR of children and adults**

Annual Joint Bleed Prevalence			Haemophilia A		Haemophilia B	
			Age < 18 (n=80)	Age ≥ 18 (n=157)	Age < 18 (n=17)	Age ≥ 18 (n=19)
Annual Joint Bleed Rate	All joints	Median (IQR)	0.0 (0.0;1.0)	1.0 (0.0;4.4)	0.0 (0.0;2.0)	0.0 (0.0;3.5)
Joint Bleed Prevalence	All joints	n (%)	26 (32.5)	94 (59.9)	8 (47.1)	8 (42.1)
	Right Ankle	n (%)	2 (2.5)	27 (17.2)	1 (5.9)	2 (10.5)
	Left ankle	n (%)	5 (6.3)	35 (22.3)	5 (29.4)	2 (10.5)
	Right knee	n (%)	13 (16.3)	27 (17.2)	1 (5.9)	2 (10.5)
	Left knee	n (%)	7 (8.8)	24 (15.3)	1 (5.9)	2 (10.5)
	Right elbow	n (%)	6 (8.0)	29 (18.5)	1 (5.9)	3 (15.8)
	Left elbow	n (%)	4 (5.0)	35 (22.3)	1 (5.9)	2 (10.5)

2 Joint bleed prevalence (%): Numerator = number of patients who had bleeds, Denominator = total cohort number,

3

For peer review only - http://bmjopen.bmj.com/site/about/guidelines.xhtml

Haemophilia joint health score

HJHS categorised by age, haemophilia type and joint are presented in Table 3. Median (IQR) of HJHS in children were 0.0 (0.0; 0.0) in both haemophilia A and B. In adults the total HJHS were higher than in children; the total HJHS is higher in haemophilia A than haemophilia B. At an individual joint level median (IQR) ankle HJHS of 4.0 (0.0; 8.0) were higher than for the knee 2.9 (4.1)/ 1.00 (0.0; 5.0) and elbow 3.3 (4.1)/ 1.0 (0.0; 7.0).

Table 3. Haemophilia joint health scores for children and adults

Haemophilia joint health scores	Haemophilia A		Haemophilia B	
Median (IQR)	Age < 18 (n=80)	Age ≥ 18 (n=157)	Age < 18 (n=17)	Age ≥ 18 (n=19)
**All Joints**	0.0 (0.0;0.0)	18.0 (6.0;31.0)	0.0 (0.0;0.0)	11.0 (5.0;24.0)
**Right ankle**	0.0 (0.0;0.0)	4.0 (0.0;8.0)	0.0 (0.0;0.0)	2.0 (0.0;7.0)
**Left ankle**	0.0 (0.0;0.0)	4.0 (0.0;8.0)	0.0 (0.0;0.0)	4.0 (1.0;8.0)
**Right knee**	0.0 (0.0;0.0)	1.0 (0.0;4.0)	0.0 (0.0;0.0)	0.0 (0.0;1.0)
**Left knee**	0.0 (0.0;0.0)	1.00 (0.0;5.0)	0.0 (0.0;0.0)	0.0 (0.0;2.0)
**Right elbow**	0.0 (0.0;0.0)	1.0 (0.0;7.0)	0.0 (0.0;0.0)	0.0 (0.0;1.0)
**Left elbow**	0.0 (0.0;0.0)	1.0 (0.0;6.0)	0.0 (0.0;0.0)	0.0 (0.0;1.0)

HJHS: Global Gait score not included

For peer review only

For peer review only - http://bmjopen.bmj.com/site/about/guidelines.xhtml

Discussion:

In this study we report the current prevalence of haemarthrosis in children and adults with severe haemophilia without current inhibitors, and associated HJHS as a measure of joint disease. The study was conducted retrospectively, using data from 2018 in a national database. In a national cohort of 2338 individuals, 463 patients had electronically-recorded fully itemised HJHS, with the sample size further reduced to 273 patients who met the fully Haemtrack compliant criteria. During the data collection period, 62% of the national cohort used Haemtrack, 20% of whom fulfilled compliance criteria set by the NHD, permitting analysis of haemarthrosis and joint health status of a representative sample of UK with severe haemophilia without inhibitors. The sample size whilst small is focused only the most compliant of patients and provides insight to the current compliance rates and reporting of joint diseases to the NHD. The results presented in this paper represent the likely best case scenario for the most complaint cases and this further highlight the 80% of patients who fail to either record or comply with treatment and raises questions as to the real compliance and adherence to treatment, as well as the concurrent joint disease in patients who do not meet the 75% NHD inclusion threshold.

In children with severe haemophilia, average AJBR were low across haemophilia types. One in three children did however experience a joint bleed during the 12 month data collection period. The majority of those included would have typically been provided prophylaxis from an early age and continue to adhere to a prophylaxis regime, but 30% of children still experienced haemarthrosis during the 12 month data collection period. HJHS itemised by joint were very low in children (Table 3) suggesting either minimal joint disease or that the HJHS might not be sensitive to early joint changes following haemarthrosis. Reliability of the HJHS has been explored in children and young adults and is reported to be sensitive to early joint changes [24, 25] , although individual joint HJHS of less than three at the knee and ankle are less able to identify pathological joint change when compared to MRI and US imaging [18]. Similarly in children, correlations between the HJHS and the Haemophilia Early Arthropathy Detection

For peer review only - http://bmjopen.bmj.com/site/about/guidelines.xhtml

with UltraSound (HEAD-US) have shown good correlations in the identification of joint pathology at the elbows and knees, however at the ankles significant difference are reported between HJHS and HEAD-US scores with underreporting of ankle joint pathology in both instances [26]. Therefore a combined approach to joint health assessment may identify pathology especially at the ankle joint prior to the progression to haemarthropathy. Canine, mouse and human in-vitro models have demonstrated chondrocyte apoptosis and reduced proteoglycan synthesis affecting cartilage matrix turnover within 48-96 hours of an induced joint bleed, suggesting a single joint bleed may have detrimental effects on joint cartilage [27-29]. Formally reported bleed rates in the NHD are relatively low, however micro bleeding (subclinical bleeding not clinically detectable, or experienced by the patient) is an emerging theme in haemophilia. Episodes of subclinical bleeding may contribute to the deterioration of joint health despite no clinically detectable signs of a joint bleed, therefore point of care ultrasound tools such as the HEAD-US may provide early evidence of joint disease [3].

In the adult population, AJBR were higher than those reported in children, with mean (SD) AJBR of 3.9 (7.0) and median (IQR) 1.0 (0.0-4.4) in haemophilia A and 2.0 (3.6) and 0.0 (0.0-3.5) in haemophilia B, respectively. The 12 month prevalence was also higher, with 60% and 41% of adults with haemophilia A and B, respectively, experiencing at least one bleed over the period. HJHS scores at the ankle joint were similar to the elbows, with knees slightly less affected. Interestingly the median scores at both the knee and elbow were lower than that of the ankle, suggesting that there is worse ankle joint health overall when compared to other joints. Ankle joint changes are driven by the mechanical demand on the ankle and forces exerted on the joint during activities of daily living, in combination with structural and functional changes often seen in adolescents and adults with severe haemophilia [30, 31]. Our data suggest that very early signs of joint disease might not be detected by the HJHS; rather it measures the cumulative effect of haemarthropathy, not detectable until later years.

AJBR in this study are slightly lower (Table 2) than those reported in the UK THUNDER study conducted three years earlier using the same NHD database [11]. Scott et al. reported a

For peer review only - http://bmjopen.bmj.com/site/about/guidelines.xhtml

median AJBR of 0.0 in children (0-11 years), 1.0 in adolescents (12-18 years) and 3.0 in adults aged 19 and above. Our prevalence data (Table 1) for both children and adults indicate a slight decrease AJBR since the Scott et al. study [16]. In terms of the treatment profile of those included in our study, about one quarter were now using an EHL product and 96% of those sampled are receiving and are compliant with treatment. In addition Scott et al did not include those patients with haemophilia B who are reported to have better joint health and less frequent joint bleeds [32]. A longitudinal evaluation of tailored frequency-escalated prophylaxis in a Canadian cohort of children aged 1.0 - 2.5 years (n=36) followed up over 10.2 years (IQR 8.5-13.6) reported median index annual haemarthrosis rates of 0·95 (0·44–1·35) which is similar to our own results. Prophylaxis treatment in Canada was driven by bleed incidence and escalated accordingly, so their treatment was more targeted and reactive [33]. The Canadian study shows that avoidance of all joint bleeding is unlikely to be possible, and in our own cohort the mean (SD) AJBR of 0.81 (1.68) and 1.00 (1.18) in haemophilia A and B children respectively, indicate that bleeding is occurring in some children even when compliant with prophylaxis. In a Dutch study of haemophiliac adults (n=62) over a 5-10 year period with a low median AJBR (IQR) 0.0 (0.0-2.0) there was still a worsening of joint health, with a HJHS increase of more than 4 points over the study period in 37.1% of patients, and with the ankle joints most often affected (30.6%) [34]. Those adults sampled in this study still had up to four joint bleeds over a 12 month period, with 60% of all adults reporting a minimum of one joint bleed. Forty percent of individuals sampled reported no bleeds and were well controlled, but for the remaining 60% it is unclear why joint bleeding occurred. Understanding why the 60% in this cohort reported haemarthrosis may lead to better targeted and individualised treatment and identification of other contributing factors such as lifestyle and altered, combined and individual joint biomechanics of the upper and lower limbs.

A limitation of this study is the low proportion of patients registered on the UK database that had full Haemtrack and itemised HJHS data recorded at the time of data collection. The NHD does not report bleed level data on patients who do not use Haemtrack owing to the difficulty

For peer review only

in collecting data from paper diaries and established links at each haemophilia centre through the NHD Haemophilia Centre Information System (HCIS), limiting analysis to Haemtrack compliant users [20]. Bias may have been introduced by the study design through the inability to include those not recording treatment in Haemtrack and those for whom HJHS examinations were not reported or itemised by joint to the NHD. Although this is the largest reported dataset of HJHS, the lack of linkage between elements of the data limits its wider utility. As electronic reporting of HJHS to the NHD becomes more routine and the dataset expands, we will be able link HJHS and joint health to rates of haemarthrosis. Haemtrack data compliance is defined as ≥75% of home delivery treatment received being recorded as used by the patient and so those who met the inclusion criteria are regarded as "good reporters" and deemed likely to be compliant with treatment [20]. The current bleeding and joint disease profiles of those who receive and record treatment, but fall below the 75% treatment adherence criteria is unknown.

Access to individual treatment dose and trough levels were not available from the database and is acknowledged as a limitation of this study. Reporting of these data relies on haemophilia centres uploading real time data, including trough levels and up to date measurements of weight but requires access to patient's data and requires better reporting methods to be achievable. Understanding joint haemarthrosis in this subset of patients may provide further insight into the real-world prevalence of haemarthrosis. This study focusses, for databasing reasons, on the most compliant cases and therefore those within the broader haemophilia population likely to be suffering the fewest consequences. It might be reasonable to expect that over the 12 month study period, comparable patients who do not report or full comply with treatment may have had higher bleed rates. Consequently it would also be expected that joint health may also be worse or deteriorating at a faster rate. Compliance is important because it represents a gap between the availability of best treatment and impact of treatment on the consequences. Less compliant patients may require different behavioural or system-based approaches to encourage compliance and better reporting and monitoring.

For peer review only

For peer review only - http://bmjopen.bmj.com/site/about/guidelines.xhtml

As expected due to the lower prevalence, the sample of haemophilia B patients in this analysis is smaller than the haemophilia A cohort, and therefore differences in joint bleed prevalence and HJHS between patients with haemophilia A and B should be interpreted with caution. Those with haemophilia B may present with a milder bleeding phenotype than that of haemophilia A regardless of severity or treatment [32, 35, 36]. In addition people with haemophilia B may display less severe levels of haemarthropathy, with differences in the specific pathophysiological mechanisms of joint disease underlined by different rates of joint deterioration and severity [37]. Direct comparison between disease types is limited and therefore further research is needed to explore whether the lower bleed rates and better joint health in people with haemophilia B suggested in this study can be confirmed.

History of spontaneous and traumatic bleeding could not be separated, owing to data reporting methods within Haemtrack. Whilst prophylaxis protects against spontaneous bleeding there is still a proportion of these treatment compliant adults reporting up to four joint bleeds in the 12 month study period. Haemarthrosis may occur as individual joint events, but our data highlights the burden on overall joint disease. A previous history of developing inhibitors and a history of on-demand treatment now using secondary prophylaxis may predispose patients to higher levels of joint disease and greater risk of subsequent haemarthrosis [11]. Further research is required therefore to understand the bleeding profile and burden of disease in adults with established joint disease and previous inhibitor status.

A further limitation is between-centre variability in HJHS assessment [38]. HJHS data from different haemophilia centres may be subject to inter-centre scoring variability, although workshops have been conducted in the UK to decrease inter-centre variability in HJHS scoring. Furthermore, we are unable to confirm the influence of other factors such as the presence of co-morbid musculoskeletal conditions on HJHS data. UKHCDO NHD data was also requested from those with moderate disease but there was insufficient data to include in the analysis. Future comparison by disease severity (severe and moderate) may provide further insight of those most at risk of haemarthropathy.

For peer review only - http://bmjopen.bmj.com/site/about/guidelines.xhtml

Clinical implication and conclusion:

In a UK cohort of Haemtrack compliant patients with severe haemophilia and without a current inhibitor, only 70% of children and 30% of adults remained haemarthrosis free during 2018. Haemarthrosis was most likely to be reported in the knee joint in children with haemophilia A, the ankle joint in children with haemophilia B, the elbow and ankle joint in adults with haemophilia A, and the elbow joint in adults with haemophilia B. Overall higher HJHS were reported for the ankle joint compared to the knee and elbow, suggesting that the ankle joint is the most severely compromised joint in people with haemophilia.

Investigation of impact on function and potential interventions that lessen the burden of disease are warranted. Future clinical studies would also benefit from understanding the bleeding profiles of those who do not meet compliance criteria for Haemtrack or other database-linked bleed data to obtain the true prevalence of haemarthrosis and joint disease.

For peer review only - http://bmjopen.bmj.com/site/about/guidelines.xhtml

Abbreviations

ABR: Annual bleed rate

AJBR: Annual joint bleed rate

CFC: Clotting factor concentrate

EHL: Extended half life

HJHS: Haemophilia joint health score

NHD: National Haemophilia Database

SHL: Standard half-life

UKHCDO: United Kingdom Haemophilia Doctors Organisation

For peer review only

For peer review only - http://bmjopen.bmj.com/site/about/guidelines.xhtml

Declarations

Ethics approval and consent to participate

Ethical approval was obtained to allow access the National Haemophilia Database, anonymised data. This study was approved by London Queen Square Research Ethics Committee (16/LO/2251) and NHS Health research Authority (IRAS ID 206141). Individual participant consent was not applicable.

Consent for publication

Consent to publish this paper was obtained from the UKHCDO NHD.

Availability of data and materials

The data that support the findings of this study are available from The National Haemophilia Database (NHD) but restrictions apply to the availability of these data, which were used under license for the current study, and so are not publicly available. Data are however available from the corresponding author upon reasonable request and with permission of the NHD.

Competing interests

RAW has received registration fees and support for travel from Roche.

DS has received research funding from Sobi, CSL and Roche; consultancy and speakers fees from Sobi and Takeda

EH has received speaker fees from Roche, sponsorship for travel from Sobi.

MJS has received research funding from Bayer; consultancy and speakers fees from Sobi and Roche; registration fees and support for travel from Sobi, Pfizer and CSL.

HJS is a HEE/NIHR Senior Clinical lecturer and has received funding from NIHR who also funded this research.

ACR is a NIHR Senior Investigator and has received funding from NIHR who also funded this research.

GJC, RW, HX, BP and MR report no competing interests

For peer review only - http://bmjopen.bmj.com/site/about/guidelines.xhtml

Funding

RAW is funded by a National Institute for Health Research (NIHR) Clinical Doctoral Research Fellowship (ICA-CDRF-2015-01-0120); ACR is in part supported through the NIHR Leeds Biomedical Research Centre, Leeds, UK. This paper presents independent research funded by the National Institute for Health Research (NIHR). The views expressed are those of the authors and not necessarily those of the NHS, the NIHR or the Department of Health.

Author's contributions

The study was conceived by RAW, AR, GC, RW and HJS. Analysis was undertaken by members of staff at the NHD (HX and BP).The manuscript was written by RAW and DS. Subsequent drafts were edited and approved by RAW, DS, MJS, AR, GC, MR, EH, HJS, and RW.

For peer review only

Acknowledgements

The authors would like to thank the centre directors, physiotherapists and staff of all UK haemophilia centres for their hard work in collecting data and responding to data queries. We would also like to thank the members of the UKHCDO Data Analysis Group for advising on data interpretation; Prof Charles Hay, Prof Peter Collins, Dr Elizabeth Chalmers, Dr Ryan Cheal, Prof Pratima Chowdary, Simon Fletcher, Dr Georgina Hall, Dr Dan Hart, Dr Ri Liesner, Andrew McNally, Ben Palmer, Paul Sartain, Dr Martin Scott, Dr Susie Shapiro, David Stephensen, Dr Hua Xiang. The staff at the National Haemophilia Database are also thanked for their contribution to data collection.

Author's information

ORCID (those with ORCID IDs)

Richard Wilkins: 0000-0003-1885-5472

Anthony Redmond: 0000-0002-8709-9992

1 Heidi Siddle: 0000-0002-6015-332X

2 Graham Chapman: 0000-0003-3983-6641

3 Martin Scott: 0000-0002-0854-1309

4 David Stephensen: 0000-0002-6175-3343

5 Rebecca Walwyn: 0000-0001-9120-1438

For peer review only

For peer review only - http://bmjopen.bmj.com/site/about/guidelines.xhtml

References

1. Bolton-Maggs, P. and J. Pasi, *Haemophilias a and b.* The Lancet, 2003. **361**(9371): p. 1801-1809.
2. Biggs, R. and R. Macfarlane, *Haemophilia and related conditions: a survey of 187 cases.* British journal of haematology, 1958. **4**(1): p. 1-27.
3. Gringeri, A., B. Ewenstein, and A. Reininger, *The burden of bleeding in haemophilia: is one bleed too many?* Haemophilia, 2014. **20**(4): p. 459-463.
4. Van Vulpen, L., K. Holstein, and C. Martinoli, *Joint disease in haemophilia: Pathophysiology, pain and imaging.* Haemophilia, 2018. **24**: p. 44-49.
5. Roosendaal, G. and F. Lafeber, *Pathogenesis of haemophilic arthropathy.* Haemophilia, 2006. **12**(s3): p. 117-121.
6. Hanley, J., et al., *Guidelines for the management of acute joint bleeds and chronic synovitis in haemophilia.* Haemophilia, 2017.
7. Manco-Johnson, M.J., et al., *Prophylaxis versus episodic treatment to prevent joint disease in boys with severe hemophilia.* New England Journal of Medicine, 2007. **357**(6): p. 535-544.
8. Oldenburg, J., *Optimal treatment strategies for hemophilia: achievements and limitations of current prophylactic regimens.* Blood, 2015. **125**(13): p. 2038-44.
9. Gringeri, A., et al., *A randomized clinical trial of prophylaxis in children with hemophilia A (the ESPRIT Study).* Journal of Thrombosis and Haemostasis, 2011. **9**(4): p. 700-710.
10. Srivastava, A., et al., *Guidelines for the management of hemophilia.* 2013. **19**(1): p. e1-e47.
11. Collins, P., et al., *Efficacy and safety of secondary prophylactic vs. on-demand sucrose-formulated recombinant factor VIII treatment in adults with severe hemophilia A: results from a 13-month crossover study.* Journal of Thrombosis and Haemostasis, 2010. **8**(1): p. 83-89.
12. Skinner, M.W., et al., *Achieving the unimaginable: Health equity in haemophilia.* Haemophilia, 2019.
13. Skinner, M.W., *WFH: closing the global gap–achieving optimal care.* Haemophilia, 2012. **18**: p. 1-12.
14. Ebbert, P.T., et al., *Emicizumab prophylaxis in patients with haemophilia A with and without inhibitors.* Haemophilia, 2020. **26**(1): p. 41-46.
15. Chowdary, P., *Extended half-life recombinant products in haemophilia clinical practice–Expectations, opportunities and challenges.* Thrombosis Research, 2019.
16. Scott, M.J., et al., *Treatment regimens and outcomes in severe and moderate haemophilia A in the UK: The THUNDER study.* Haemophilia, 2019. **25**(2): p. 205-212.
17. Berntorp, E., et al., *European retrospective study of real-life haemophilia treatment.* Haemophilia, 2017. **23**(1): p. 105-114.
18. Poonnoose, P., et al., *Correlating clinical and radiological assessment of joints in haemophilia: results of a cross sectional study.* Haemophilia, 2016. **22**(6): p. 925-933.
19. Thornburg, C.D., N.A.J.P.p. Duncan, and adherence, *Treatment adherence in hemophilia.* Patient preference adherence, 2017. **11**: p. 1677.
20. Hay, C., et al., *The haemtrack home therapy reporting system: Design, implementation, strengths and weaknesses: A report from UK Haemophilia Centre Doctors Organisation.* Haemophilia, 2017. **23**(5): p. 728-735.

For peer review only - http://bmjopen.bmj.com/site/about/guidelines.xhtml

21. UKHCDO. *united kingdom haemophilia centre doctors organisation*. 2020 [cited 2020 05/04/2020]; Available from: http://www.ukhcdo.org/nhd/.
22. UKHCDO, *UKHCDO Annual Report 2019, including bleeding disorder statistics* 2019.
23. Richards, M., et al., *A United Kingdom Haemophilia Centre Doctors' Organization guideline approved by the British Committee for Standards in Haematology: guideline on the use of prophylactic factor VIII concentrate in children and adults with severe haemophilia A.* British journal of haematology, 2010. **149**(4): p. 498-507.
24. Boehlen, F., L. Graf, and E. Berntorp, *Outcome measures in haemophilia: a systematic review.* European Journal of Haematology, 2014. **93**: p. 2-15.
25. Hilliard, P., et al., *Hemophilia joint health score reliability study.* Haemophilia, 2006. **12**(5): p. 518-525.
26. Aspdahl, M., et al., *Comparison of joint status in children with haemophilia A using ultrasound and physical examination.* European Journal of Physiotherapy, 2018. **20**(3): p. 172-177.
27. Hakobyan, N., et al., *Pathobiology of hemophilic synovitis I: overexpression of mdm2 oncogene.* Blood, 2004. **104**(7): p. 2060-2064.
28. Roosendaal, G., et al., *Blood‐induced joint damage: a canine in vivo study.* Rheumatism: Official Journal of the American College of Rheumatology, 1999. **42**(5): p. 1033-1039.
29. Hooiveld, M., et al., *Blood-induced joint damage: longterm effects in vitro and in vivo.* The Journal of rhematology, 2003. **30**(2): p. 339-344.
30. Pasta, G., et al., *Orthopaedic management of haemophilia arthropathy of the ankle.* Haemophilia, 2008. **14**(s3): p. 170-176.
31. Rodriguez-Merchan, E.C., *Management of hemophilic arthropathy of the ankle.* Cardiovascular and Hematological Disorders - Drug Targets, 2017. **17**(2): p. 111-118.
32. Mannucci, P. and M. Franchini, *Is haemophilia B less severe than haemophilia A?* Haemophilia, 2013. **19**(4): p. 499-502.
33. Feldman, B.M., et al., *Tailored frequency-escalated primary prophylaxis for severe haemophilia A: results of the 16-year Canadian Hemophilia Prophylaxis Study longitudinal cohort.* Lancet Haematol, 2018. **5**(6): p. e252-e260.
34. Kuijlaars, I.A.R., et al., *Monitoring joint health in haemophilia: Factors associated with deterioration.* Haemophilia, 2017. **23**(6): p. 934-940.
35. Santagostino, E., et al., *Severe hemophilia with mild bleeding phenotype: molecular characterization and global coagulation profile.* Journal of Thrombosis and Haemostasis, 2010. **8**(4): p. 737-743.
36. Castaman, G. and D.J.h. Matino, *Hemophilia A and B: molecular and clinical characteristics of similar, but different diseases.* Haematologica, 2019: p. haematol. 2019.221093.
37. Melchiorre, D., et al., *Clinical, instrumental, serological and histological findings suggest that hemophilia B may be less severe than hemophilia A.* Haematologica, 2016. **101**(2): p. 219-225.
38. Nijdam, A., et al., *Using routine Haemophilia Joint Health Score for international comparisons of haemophilia outcome: standardization is needed.* Haemophilia 2016. **22**(1): p. 142-147.

For peer review only - http://bmjopen.bmj.com/site/about/guidelines.xhtml

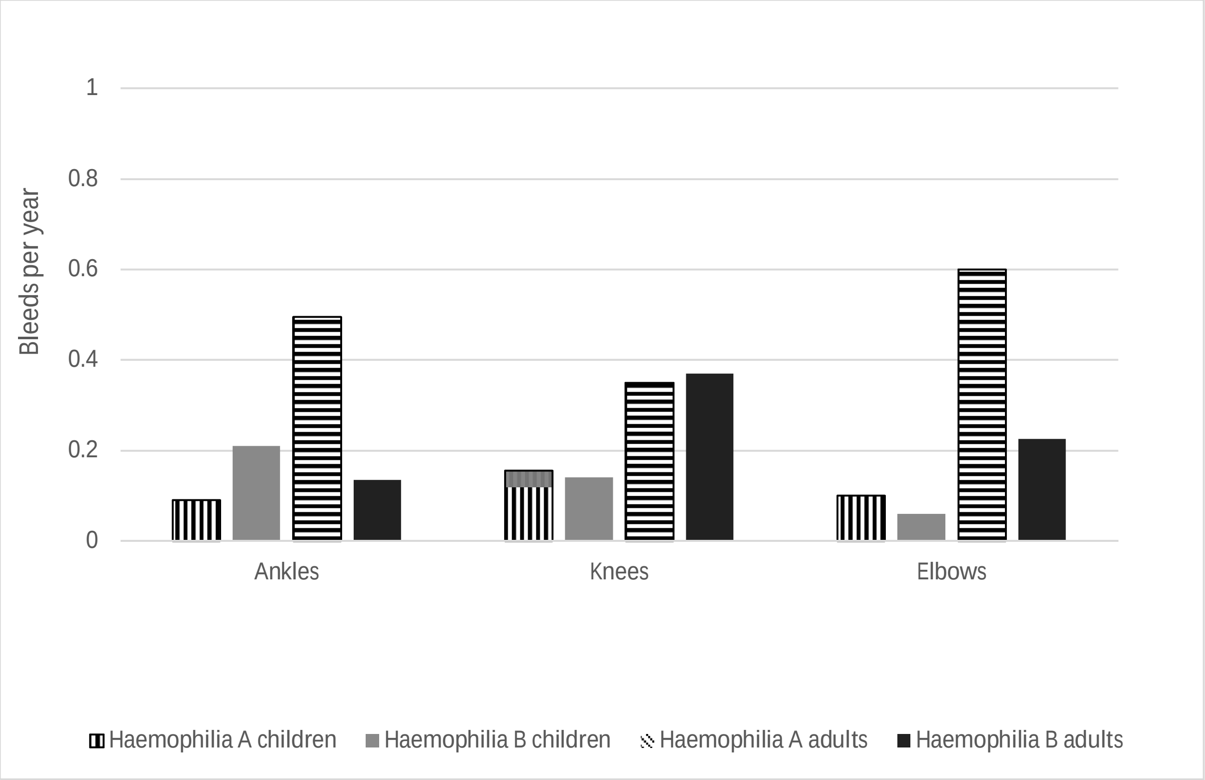

For peer review only

STROBE Statement

Checklist of items that should be included in reports of observational studies

Section/Topic	Item No	Recommendation	Reported on Page No
**Title and abstract**	1	(*a*) Indicate the study's design with a commonly used term in the title or the abstract	3
		(*b*) Provide in the abstract an informative and balanced summary of what was done and what was found	3
**Introduction**			
Background/rationale	2	Explain the scientific background and rationale for the investigation being reported	5-6
Objectives	3	State specific objectives, including any prespecified hypotheses	7
**Methods**			
Study design	4	Present key elements of study design early in the paper	8
Setting	5	Describe the setting, locations, and relevant dates, including periods of recruitment, exposure, follow-up, and data collection	8-9
Participants	6	(*a*) *Cohort study*—Give the eligibility criteria, and the sources and methods of selection of participants. Describe methods of follow-up<br>*Case-control study*—Give the eligibility criteria, and the sources and methods of case ascertainment and control selection. Give the rationale for the choice of cases and controls<br>*Cross-sectional study*—Give the eligibility criteria, and the sources and methods of selection of participants	7-9
		(*b*) *Cohort study*—For matched studies, give matching criteria and number of exposed and unexposed<br>*Case-control study*—For matched studies, give matching criteria and the number of controls per case	n/a
Variables	7	Clearly define all outcomes, exposures, predictors, potential confounders, and effect modifiers. Give diagnostic criteria, if applicable	7,8
Data sources/measurement	8*	For each variable of interest, give sources of data and details of methods of assessment (measurement). Describe comparability of assessment methods if there is more than one group	7,8
Bias	9	Describe any efforts to address potential sources of bias	8,9
Study size	10	Explain how the study size was arrived at	10
Quantitative variables	11	Explain how quantitative variables were handled in the analyses. If applicable, describe which groupings were chosen and why	9
Statistical methods	12	(*a*) Describe all statistical methods, including those used to control for confounding	9
		(*b*) Describe any methods used to examine subgroups and interactions	9
		(*c*) Explain how missing data were addressed	n/a
		(*d*) *Cohort study*—If applicable, explain how loss to follow-up was addressed<br>*Case-control study*—If applicable, explain how matching of cases and controls was addressed<br>*Cross-sectional study*—If applicable, describe analytical methods taking account of sampling strategy	n/a
		(*e*) Describe any sensitivity analyses	n/a

For peer review only - http://bmjopen.bmj.com/site/about/guidelines.xhtml

Section/Topic	Item No	Recommendation	Reported on Page No
**Results**			
Participants	13*	(a) Report numbers of individuals at each stage of study—eg numbers potentially eligible, examined for eligibility, confirmed eligible, included in the study, completing follow-up, and analysed	10
		(b) Give reasons for non-participation at each stage	n/a
		(c) Consider use of a flow diagram	n/a
Descriptive data	14*	(a) Give characteristics of study participants (eg demographic, clinical, social) and information on exposures and potential confounders	
		(b) Indicate number of participants with missing data for each variable of interest	10
		(c) *Cohort study*—Summarise follow-up time (eg, average and total amount)	n/a
Outcome data	15*	*Cohort study*—Report numbers of outcome events or summary measures over time	n/a
		Case-control study—Report numbers in each exposure category, or summary measures of exposure	n/a
		Cross-sectional study—Report numbers of outcome events or summary measures	n/a
Main results	16	(*a*) Give unadjusted estimates and, if applicable, confounder-adjusted estimates and their precision (eg, 95% confidence interval). Make clear which confounders were adjusted for and why they were included	10
		(*b*) Report category boundaries when continuous variables were categorized	
		(*c*) If relevant, consider translating estimates of relative risk into absolute risk for a meaningful time period	
Other analyses	17	Report other analyses done—eg analyses of subgroups and interactions, and sensitivity analyses	10
**Discussion**			
Key results	18	Summarise key results with reference to study objectives	11
Limitations	19	Discuss limitations of the study, taking into account sources of potential bias or imprecision. Discuss both direction and magnitude of any potential bias	13
Interpretation	20	Give a cautious overall interpretation of results considering objectives, limitations, multiplicity of analyses, results from similar studies, and other relevant evidence	11,12
Generalisability	21	Discuss the generalisability (external validity) of the study results	13
**Other Information**			
Funding	22	Give the source of funding and the role of the funders for the present study and, if applicable, for the original study on which the present article is based	21

**Give information separately for cases and controls in case-control studies and, if applicable, for exposed and unexposed groups in cohort and cross-sectional studies.*

Note: An Explanation and Elaboration article discusses each checklist item and gives methodological background and published examples of transparent reporting. The STROBE checklist is best used in conjunction with this article (freely available on the Web sites of PLoS Medicine at http://www.plosmedicine.org/, Annals of Internal Medicine at http://www.annals.org/, and Epidemiology at http://www.epidem.com/). Information on the STROBE Initiative is available at www.strobe-statement.org.

For peer review only - http://bmjopen.bmj.com/site/about/guidelines.xhtml
